# Effect of surrounded shade and specimen`s thickness on color adjustment potential of a single-shade composite

**DOI:** 10.1590/0103-6440202204973

**Published:** 2022-10-21

**Authors:** Mariana Silva Barros, Paula Fernanda Damasceno Silva, Márcia Luciana Carregosa Santana, Rafaella Mariana Fontes de Bragança, André Luis Faria-e-Silva

**Affiliations:** 1 Graduate Program in Dentistry, Federal University of Sergipe, Aracaju, SE, Brazil

**Keywords:** Color adjustment potential, Color blending, Resin composite, Opacity

## Abstract

This study evaluated the effect of surrounded shade and specimens` thickness on the color adjustment potential (CAP) of a single-shade composite. The composite Vittra APS Unique was surrounded (dual specimens) or not (simple specimens) by a control composite (shade A1, A2, or A3). Simple specimens of the control composite were also confectioned. Opacity and whiteness index for dentistry (WI_D_) were calculated for simple specimens. Color differences between the simple (ΔE*_SIMPLE_)/ dual specimens (ΔE*_DUAL_) and the controls were calculated. CAP was calculated based on the ratio between ΔE* _SIMPLE_ and ΔE* _DUAL_. The tested composite presented lower opacity (53 to 62% vs. 80 to 93%) and higher WI_D_ (≈ 42 vs. 18 to 32) than controls. Irrespective of the specimens’ thickness (1.0/ 1.5 mm), the lowest values of ΔE* _SIMPLE_ (11.1/ 10.8) and ΔE*_DUAL_ (7.2/ 6.1) were observed using the surrounding shade A1. The shade A3 yielded higher ΔE*_SIMPLE_ (16.4/ 17.1) and ΔE* _DUAL_ (11.3/ 12.3) than the A2 (ΔE*_SIMPLE_ = 13.4/ 14.6; and ΔE* _DUAL_ = 9.7/ 10.3). The specimen`s thickness significantly affected the CAP (0.35 and 0.44 for 1.0 and 1.5 mm, respectively) only for shade A1, which had the highest CAP values. The shade A3 resulted in higher CAP values (0.31) than A2 (0.27) when 1.0-mm thick specimens were used, but similar values were observed for 1.5 thick specimens (≈ 0.29). In conclusion, both surrounding shade and specimen thickness can affect the CAP of a single-shade resin composite.

## Resumo

Este estudo avaliou o efeito da cor circundante e da espessura da amostra no potencial de ajuste de cor (PAC) de uma resina monocromática. O compósito Vittra APS Unique foi circundado (amostras duplas) ou não (amostras simples) com um compósito controle (cor A1, A2 ou A3). Amostras simples do compósito controle também foram confeccionadas. A opacidade e o índice de brancura para odontologia (IB_O_) foram calculados para as amostras simples. As diferenças de cor entre amostras simples (ΔE*_SIMPLE_) / duplas (ΔE*_DUAL_) e os controles foram calculados. PAC foi calculado baseado na razão entre ΔE* _SIMPLE_ e ΔE* _DUAL_. O compósito testado apresentou menor opacidade (53 a 62% vs. 80 a 93%) e maior IB_O_ (≈ 42 vs. 18 a 32) que os controles. Independente da espessura da amostra (1,0/ 1,5 mm), os valores mais baixos de ΔE* _SIMPLE_ (11,1/ 10,8) e ΔE*_DUAL_ (7,2/ 6,1) foram observadas quando a resina circundante era A1. A cor A3 resultou em maior ΔE*_SIMPLE_ (16,4/ 17,1) e ΔE* _DUAL_ (11,3/ 12,3) do que A2 (ΔE*_SIMPLE_ = 13,4/ 14,6; e ΔE* _DUAL_ = 9,7/ 10,3). A espessura das amostras afetou significativamente o PAC (0,35 e 0,44 para 1,0 e 1,5 mm, respectivamente) apenas para a cor A1, que teve os maiores valores de PAC. A cor A3 resultou nos maiores valores de PAC (0,31) que A2 (0,27) quando amostras de 1,0 mm de espessura foram usadas, mas valores similares foram observados para amostras de 1,5 mm (≈ 0,29). Como conclusão, tanto a cor circundante como a espessura das amostras podem afetar o PAC de um compósito resinoso monocromático.

## Introduction

Esthetic dental restorations depend on an adequate matching between the color of restorative material and that observed in tooth structures. Optical phenomena such as diffusion, scattering, absorption, and light reflection interacting with the dentin and enamel are responsible for the dental color ^1^ ^,^ ^2^ ^,^ ^3^. The intrinsic color of teeth is associated with the scattering and absorption of different wavelengths in dentinal tubules and enamel hydroxyapatite crystals ^2^ ^,^ ^3^. Thus, the ultimate color is related to the thicknesses of dental substrates and their curvatures, modifying the direction of the reflected light ^4^.

The complexity of optical phenomena challenges the clinicians in mimicking the tooth color using direct resin composites. Most of the composites` manufacturers provide their systems are based on materials with different translucency levels. Further to a proper shade selection, an adequate relation between the composite translucency and the thickness of its layer strongly affects the esthetic result ^4^ ^,^ ^5^ ^,^ ^6^. Another factor related to color perception is the illuminant since an object's color changes under different light sources ^7^. Therefore, the experience of clinicians to select the composite shades and build the restorations up are crucial factors, improving the predictability of the restorative procedure ^8^ ^,^ ^9^.

Composites with improved ability to shift their color toward adjacent tooth structures can facilitate restorative procedures. The “color shifting” or “color adjustment potential” (CAP) of composite results from both blending effect (visual and subjective perception) and its translucency ^10^ ^,^ ^11^ ^,^ ^12^ ^,^ ^13^ ^,^ ^14^. Therefore, since the composite`s thickness is closely related to its translucency, it is reasonable to assume that increasing the thickness of the composite layer might reduce its ability to mimic the adjacent substrates. Moreover, the color of the adjoining substrate also might affect the CAP, but this effect is barely studied. In fact, despite its importance in esthetic dentistry, the mechanisms involved in the color adjustment of composites remain not fully understood.

 This study evaluated the effect of surrounded shade and specimens` thickness on CAP of a single-shade resin composite using an instrumental method. The study hypothesized that the surrounded shade and the specimens` thickness do not affect the CAP values.

## Material and methods

The CAP of the single-shade resin composite Vittra APS Unique (FGM, Joinville, SC, Brazil) was evaluated in the present study. The composite Forma (Ultradent, Indaiatuba, SP, Brazil) was used as the control and surrounded color (shades A1D, A2D, and A3D). Specimens were built with single (simple) and two (dual) composites. Composites from the same manufacturer tend to present similar optical behavior considering the similarity in the monomeric and filler compositions. Therefore, composites from another manufacturer were used as controls to avoid possible bias. For the simple specimens, the composites were inserted in a single increment into a metallic matrix with 8-mm of internal diameter and depth of 1.0 or 1.5 mm (n = 5). The composites were covered with a polyester strip. They were light-cured with a LED-based light unit (Radii-Cal, SDI, Victoria, Australia) used in the standard mode (1,200 mW/cm²) for the 40s. The light-curing tip was positioned approximately 2 mm from the specimen to allow the light to reach its entire surface.

**Figure 1:**
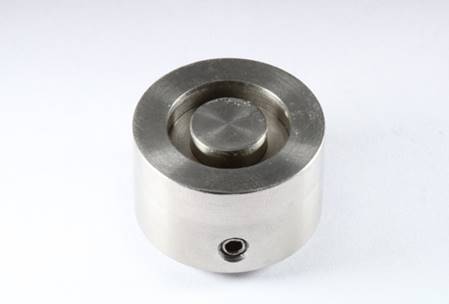
Metallic matrix used to build dual specimens.

A matrix with a 16-mm internal diameter and a metal cylinder with 8-mm in its center was used to obtain dual specimens (Figure 1). The internal depth of this matrix was adjustable to obtain specimens with either 1.0 or 1.5-mm of thickness. For each shade, the composite Forma was inserted into the matrix in a single increment and covered with a polyester strip (n = 5). Four 40s photoactivations were performed. The position of the light-curing unit tip was changed between each photoactivation to cover the entire surface of the specimen. Afterward, the central metal cylinder was moved down, leaving the correspondent space, which was filled with the composite Vittra APS Unique. This last composite was light-cured for the 40s, and the specimens were stored in a dry condition for at least seven days before the color measurements.

The specimens` color was assessed with a spectrophotometer (SP60, X-Rite, Grand Rapids, MI, USA) used in reflectance mode. The device has an aperture diameter of 8 mm, and the readings were carried out with a 2° observer angle and illuminant D65. The coordinates of the LAB system from the Commission Internationale de L’Eclairage (CIE) were recorded. This system is based on the lightness (coordinate L*) and the chromaticity coordinates a* (red-green axis) and b* (yellow-blue axis). Color coordinates were read with the specimens placed against a white background (ColorChecker grayscale, X-Rite, Grand Rapids, MI, USA). For the dual specimens, the color was measured by positioning the spectrophotometer tip on the specimen`s center corresponding to the composite Vittra APS Unique. The specimens were placed onto a paper matrix that assured the color readings in their center.

The opacity of simple specimens was also automatically calculated by the spectrophotometer based on the contrast between the colors measured against white and black backgrounds. Based on Lab data, the whiteness index for dentistry (WI_D_) of all specimens was calculated using the following formula ^15^:

$$WID=0.551\times L^{*}\;-2.324\;\times a^{*}-1.1\times b^{*}$$ Equation 1

The color differences (ΔE*_SIMPLE_) between the control composite (shades A1D, A2D, and A3D) and Vittra APS Unique using only the color of simple specimens. The following formula was used:

$${\Delta E}^{*}\;=\;{{{\left[(\Delta L^{*}\right)}^{2}+{\left(\Delta a^{*}\right)}^{2}+(\Delta b^{*})}^{2}]}^{1/2}$$ Equation 2

**Figure 2:**
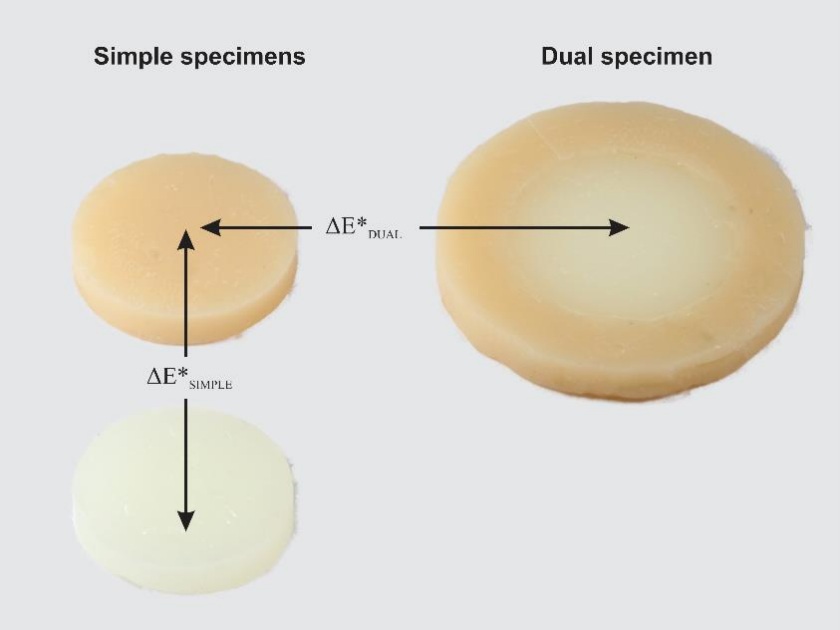
Schematic illustration of specimens` arrangement to calculate ΔE*_simple_ and ΔE*_dual_.

Furthermore, the color differences between the simples specimens of the control composite and the dual specimens with the same shade surrounding the composite Vittra APS Unique (ΔE*_DUAL_) were calculated to estimate the CAP values. Figure 2 illustrates the arrangement of the specimens to calculate both ΔE*_SIMPLE_ and ΔE*_DUAL_.

For each experimental condition (thickness vs. surrounding shade), the CAP was calculated using the following formula ^11^:

$$CAP=1-({{\Delta E}^{*}}_{DUAL}/{{\Delta E}^{*}}_{SIMPLE}\;)$$ Equation 3

The data were analyzed for normal distribution (Shapiro-Wilk test) and homogeneity of variance (Levene's test). For each outcome, data were analyzed using two-way ANOVA. The independent variables analyzed were ‘thickness’ and “composite shade’ vs. surrounding shade.’ Pair-wise comparisons were performed with Tukey's test, and a significance level of 95% was set for all analyzes.

## Results

The results for WI_D_ and opacity for simple specimens are displayed in Table 1. Two-way ANOVA showed that both factors ‘thickness’ (p < 0.001) and ‘composite shade’ (p < 0.001) affected the WI_D_ values, and the interaction between the factors was also significant (p < 0.001). Only for the shades A2 and A3, the specimens` thickness affected the WI_D_ values (1.5 mm < 1.0 mm). Irrespective of thickness, the whitest specimens were observed for Vittra APS Unique, and the whiteness increased from A3 toward A1. The opacity was also affected by specimen’s ‘thickness’ (p < 0.001), ‘composite shade’ (p < 0.001), and the interaction between the factors (p = 0.001). For both thicknesses, Vittra APS Unique showed the lowest opacity values. No differences in opacity among the shades of composite Forma were observed for 1.5-mm thick specimens. On the other hand, for specimens with 1.0mm of thickness, the shade A1 was opaquer than the others were, which did not differ between them. Opacity increased for thicker specimens.

**Table 1:** Means (standard deviations) of whiteness index and opacity for simple composite resin specimens according to the specimen`s thickness.

Outcome		WID		Opacity (%)	
Specimen`s thickness		1.0 mm	1.5 mm	1.0 mm	1.5 mm
Composite Resin	Forma A1	31.5 (0.3) ^Ba^	30.9 (0.2) ^Ba^	88.3 (1.4) ^Ab^	92.9 (2.2) ^Aa^
	Forma A2	27.0 (0.3) ^Ca^	25.3 (0.4) ^Cb^	79.8 (2.3) ^Bd^	91.6 (1.5) ^Aa^
	Forma A3	20.1 (0.5) ^Da^	18.4 (0.3) ^Db^	81.6 (2.3) ^Bb^	90.4 (1.7) ^Aa^
	Vittra APS Unique	42.2 (0.5) ^Aa^	42.1 (1.0) ^Aa^	53.0 (0.9) ^Cb^	61.6 (1.8) ^Ba^

For each outcome, distinct letters (uppercase comparing shades, lowercase comparing thicknesses) indicate statistical difference at Tukey`s test (p < 0.05). WID: whiteness index.

Table 2 displays the color difference between control shade and simple specimens of Vittra APS Unique (ΔE*_SIMPLE_) and between control shade and dual specimens of Vittra APS Unique (ΔE*_DUAL_). Two-way repeated-measures ANOVA showed that ΔE*_SIMPLE_ values were affected by both factors ‘thickness’ (p < 0.001) and ‘shade’ (p < 0.001), as well as by the interaction between the factors (p < 0.001). Values of ΔE*_DUAL_ were similarly influenced by the surrounded ‘shade’ and interaction between the factors (p < 0.001 for both). However, the ‘thickness’ alone was not significant (p = 0.179). Regardless of the thickness, the color difference increased toward more chromatic resins (from A1 to A3) for both color differences (ΔE*_SIMPLE_ and ΔE* _DUAL_). Only for shade A1, the color difference values tended to reduce as the thickness increased (no significant difference for ΔE*_SIMPLE_). The opposite was observed for the other shades (no significant difference for ΔE*_DUAL_ and A2).

**Table 2:** Color differences (standard deviation) among simple (ΔE*_simple_) and dual (ΔE*_dual_) composite resin specimens of Vittra APS Unique and the control shades.

Outcome		ΔE*_SIMPLE_		ΔE*_DUAL_	
Specimen`s thickness		1.0 mm	1.5 mm	1.0 mm	1.5 mm
Shade of outer resin	A1	11.1 (0.6) ^Ca^	10.8 (0.8) ^Ca^	7.2 (0.9) ^Ca^	6.1 (0.7) ^Cb^
	A2	13.4 (0.6) ^Bb^	14.6 (0.7) ^Ba^	9.7 (0.8) ^Ba^	10.3 (0.8) ^Ba^
	A3	16.4 (0.5) ^Ab^	17.1 (0.3) ^Aa^	11.3 (0.8) ^Ab^	12.3 (0.8) ^Aa^

For each outcome, distinct letters (uppercase comparing shades, lowercase comparing thicknesses) indicate statistical difference at Tukey`s test (p < 0.05).

**Figure 3:**
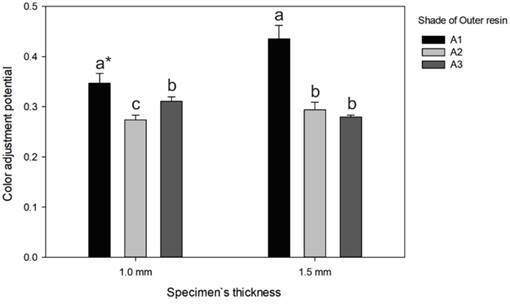
Means (standard deviation) of color adjustment potential of the composite Vittra APS Unique according to the shade of outer resin and specimen`s thickness. For each thickness, distinct letters indicate statistical difference at Tukey`s test (p < 0.05). * Statistical difference from 1.5-mm thick specimens for the same shade of outer resin.

Regarding the values of CAP, the factors ‘composite shade’ (p < 0.001) and ‘thickness’ (p = 0.010) affected the results, and the interaction between these factors was also significant (p = 0.002) - Figure 3. Irrespective of the specimen`s thickness, the highest potential was observed when the outer resin was A1. Differences between the other shades of outer resin were observed only for the thinner specimens (A2 < A3). The specimen`s thickness affected the CAP values only when the outer resin was A1 (1.5 mm > 1.0 mm).

## Discussion

In the present study, specimen thickness and surrounded shade affected the CAP values of a single-shade resin. Therefore, we rejected the null hypotheses of the study. The rationale of CAP calculation is that the tested composite shifts its color toward the surrounding composite shade ^11^ ^,^ ^12^. Therefore, compared with the control composite, it is expected that the color difference is lower for dual specimens (ΔE*_DUAL_) than for the simples (ΔE*_SIMPLE_). Consequently, the CAP values are directly related to the difference between ΔE*_SIMPLE_ and ΔE*_DUAL_.

When the color of single composite specimens was analyzed, the highest values of WI_D_ were observed for Vittra APS Unique. It is reasonable to associate these results with the fact that the Vittra APS Unique is less opaque (53 to 61%) than the composite Forma (80 to 93%), allowing enhanced visualization of the white background ^16^ ^,^ ^17^. In general, specimens thicker than 2 mm are required to prevent the background from influencing the color measurements ^18^ ^,^ ^19^. However, composites are usually inserted in layers thinner than 2 mm to restore esthetic areas such as incisal edges of cavities class IV ^20^ ^,^ ^21^. Therefore, despite some background effects on the color assessment, we choose to evaluate specimens with more clinically suitable thickness ranging from 1.0 to 1.5 mm. Moreover, it is essential to emphasize that no clinically relevant WI_D_ differences were observed by increasing the specimen`s thickness from 1.0 to 1.5 mm. This last matter suggests that the background effect was less significant than expected.

The simple specimens` readings also showed that the WI_D_ of the Vittra APS Unique was closer to that calculated to the shade A1. Consequently, the lowest values of ΔE*_SIMPLE_ were observed between simple specimens of the tested composite and Forma A1D. Moreover, comparing these last composites with dual specimens also resulted in the lowest values of ΔE*_DUAL_. Since the CAP calculation is based on relative reduction in ΔE* values, the percentage of reduction on color differences caused by placing the composite Vittra APS Unique surrounded by the compared (control) shade should be used to explain the CAP. For shade A1, the values of ΔE*_DUAL_ were 39% lower (35 and 44% for specimens with 1.0 and 1.5 mm, respectively) than those calculated for ΔE*_SIMPLE_. In contrast, a reduction of approximately 30% (ranging from 28 to 31%) was observed for the other shades. Therefore, the highest values of CAP for the Vittra APS Unique were observed when the composite was inserted into a cylinder built using the composite A1, mainly when thicker specimens were evaluated.

Further to the presence of pigments, the color of dental composites also results from its bulk structure selecting the reflection of specific wavelengths ^22^ ^,^ ^23^ ^,^ ^24^ ^,^ ^25^. Animals like butterflies and peacocks present several colors due to photonic crystals instead of pigments ^26^. A photonic crystal is an optical nanostructure that changes its refraction index periodically, affecting light propagation ^27^. This phenomenon can be obtained in dental composites by using specific sizes and shapes of filler particles that selectively reflect certain bands of wavelengths of light ^22^ ^,^ ^24^. For instance, the manufacturer of the composite Omnichroma (Tokuyama Dental, Tokyo, Japan) states that using 260nm spherical fillers generates the red-to-yellow color, which improves its ability to match the color of natural teeth ^24^.

Furthermore, it is reported that an increase in the translucency of the composite Omnichroma after its polymerization also contributes to the color-shifting ability ^14^. The manufacturer of Vittra APS Unique provides a lack of information regarding the mechanism explaining the material`s color shifting. Still, this effect is reported due to its translucency increasing after the polymerization. Indeed, this composite polymerized presented lower opacity in the present study than the others used as controls. It is also important to emphasize that the manufacturer of Vittra APS Unique recommends using an additional layer of an opaquer composite for very dark substrates. Therefore, it is reasonable to relate the CAP of Vittra APS Unique more to its high translucency than some color shifting ability. Finally, the CAP values observed were like those observed for regular composites in a prior study using a similar methodology used in the present study ^11^.

Dental composites with high CAP can facilitate clinicians obtaining satisfactory esthetic restorations and compensate for any failure on shade selection. The present study's findings showed that the blending effect of Vittra APS Unique depends on the surrounding color. The CAP values were higher when the outer composite was A1 than for darker shades, and using a thicker layer increased the blending effect only when the lighter shade was used. A limitation of the present study was that the colors were assessed only using a white background. Considering the high translucency of Vittra APS Unique, it is expected that different results might be observed by using darker backgrounds. Moreover, the results observed for the Vittra APS cannot be extrapolated to other single-shade composites since the factors affecting the color-shifting ability is material-dependent.
